# Supplementary material for: Rapid progression and short-term mortality during the January 2023 to July 2024 Cholera outbreaks in Zambia: A retrospective facility-based study
Source: PLoS One. 2025 Dec 8;20(12):e0330969. doi: 10.1371/journal.pone.0330969 (PMC12685159; doi:10.1371/journal.pone.0330969)
Supplement: Attachment — Submitted filename: Reviewers comments.docx [file pone.0330969.s004.docx]

**Response to Reviewers**

We would like to sincerely thank the Editor and the reviewers for overseeing this process and for providing constructive comments on our manuscript entitled *"Rapid Progression, Delayed Response: Survival and Mortality Patterns in Zambia’s January 2023–July 2024 Cholera Outbreak."* We have carefully addressed each concern raised and provide detailed responses below. We remain happy to make any further revisions as required.

**Reviewer Comments and Responses**

1. **Comment**: We note you have included a table to which you do not refer in the text of your manuscript. Please ensure that you refer to Table 2 and Table 5 in your text; if accepted, production will need this reference to link the reader to the Table.

**Response**: The comment has been addressed, see page 5, 8, and 10

2. **Comment:** Among the 2,162 patients included in the survival analysis (What figure was used for survival analysis? 3,655 or 2,162?). This is confusing and needs to be reconciled, as it may alter subsequent calculations.

**Response:** The issue has been resolved; see page 2. “Of the 3,655 cholera patients enrolled in the study, 2,162 patients were included in the survival analysis”

3. **Comment:** 13.9% were asymptomatic (How and why did asymptomatic patients get into treatment centers and included in any stage of analysis?).

**Response:** Although 13.9% of patients were asymptomatic, they were included because cholera surveillance often identifies contacts of confirmed cases or individuals presenting for testing during outbreaks. These patients may have sought care due to exposure concerns, mild symptoms, or routine screening. Including them allows the analysis to capture the full spectrum of reported cases and infection severity.

4. **Comment:** Cases up to December 2024 were said to have been included. This needs reconciliation.

**Response:** This was a typographical error and has been corrected; see page 5.

5. **Comment:** Patient admission status, vital status at reporting, and laboratory testing prior to notification what is this?

**Response:** The sentence has been clarified for improved flow and clarity; see page 5.

6. **Comment:** Males had an estimable 25th percentile survival time of 16 days, while all percentile estimates were unavailable for females. Why?

**Response:** Percentile survival times for females were not estimable because the number and timing of deaths were too few to reach the required proportion of events (25%, 50%, or 75%) needed for calculation.

7. **Comment:** Figure 4: Kaplan-Meier Survival Curves for Cholera Patients Stratified by Sex, Age, and Case Classification.

**Response:** Figures were generated in Stata and have been attached separately.

8. **Comment:** Although children are typically the most affected group, their outcomes were comparatively better. This may be due to targeted pediatric treatment protocols???

**Response:** The statement has been deleted; see page 13.

9. **Comment:** The findings stress the need for earlier triage, decentralized rehydration efforts (this does not emanate from the study), and targeted outreach for high-risk populations.

**Response:** The statement has been deleted; see page 13.

10. Comment: Results state 3,655 patients total; 0–14 years = 37.8%. Later, Table 2 claims the 0–14 group “constituted the largest portion of the cohort (788 patients)” for survival, but another row for 0–14 appears to show “2,285” in the “Number of Patients” column ( which exceeds both the survival cohort (2,162) and contradicts earlier totals.

Response: It was a typo it has been corrected see page 9

11. **Comment:** Report 95% CIs for incidence rates (events per 100 person-days), log-rank tests, and any hazard ratios if you add regression.
    Given that multiple risk factors are discussed (age, sex, case classification), a multivariable Cox model (or Poisson/negative binomial model with an offset for person-time) is needed to adjust for confounding.

**Response:** The log-rank tests were included on the Kaplan Meier just the next section. The Multivariable Cox regression has been included.

12. Reviewer: You report RDTs performed in 11.2% of cases, but then state ELISA was “the most commonly used method” (76%). In cholera surveillance, culture and PCR are standard confirmatory tests; the “serological (ELISA)” method requires justification, specific assay names, and denominators.

**Response:** Although culture and PCR are the standard confirmatory methods for cholera, ELISA-based serology was also used during the outbreak as part of the national surveillance protocol. ELISA tests (LOINC 44012-3) were primarily applied in settings where culture or PCR capacity was limited, providing rapid preliminary confirmation of suspected cases. Including ELISA allowed broader coverage and timely reporting in resource-constrained settings. The manuscript now specifies the assay names, LOINC codes, and denominators for all test types to ensure clarity and reproducibility.

13. Reviewer: The paper emphasizes “delays,” yet the descriptive statistic for symptom onset to first healthcare contact is median 0 days (IQR 0–2). Please present the full distribution (e.g., proportions arriving ≥24h, ≥48h) and stratify by outcome (died vs survived). If many deaths occur rapidly after arrival, the bottleneck may be in triage/initial resuscitation rather than pre-hospital delay.
14. **Comment:** This may be due to targeted pediatric treatment protocols, earlier healthcare seeking, or greater physiological resilience among younger patients.

**Response:** Wording has been revised to reflect multiple plausible factors without speculation: improved pediatric outcomes likely result from specialized treatment protocols, prompt care-seeking, and potential physiological resilience; see page 13

15. **Comment:** The speed of fatal deterioration also challenges the “7-1-7” timeliness benchmark. Please explain.

**Response:** We have clarified that “7-1-7” refers to detecting outbreaks within 7 days, notifying authorities within 1 day, and initiating an effective response within 7 days. Given cholera’s rapid progression, this benchmark may be insufficient; see page 14.

16. **Comment:** Community-based early warning systems and ORPs do not emanate from this study.
    **Response:** The statement has been deleted; see page 15.
17. **Comment:** Recommendations: There is a critical need to include a subsection with actionable, stakeholder-targeted recommendations.
    **Response:** We thank the reviewer for this important point. A new ‘Recommendations’ subsection has been added to the Discussion, providing actionable guidance for stakeholders; see page 16.
18. **Comment**: Please confirm whether there are legal or ethical restrictions on sharing your data publicly.

    If legal or ethical restrictions apply, please provide all necessary instructions and non-author contact information (preferably email) for a data access committee, ethics committee, or other institutional body that other researchers would require to request access to your data.

**Response**: Dataset has been attached.

**Closing:**
We sincerely appreciate the reviewers’ careful reading of our manuscript and their constructive feedback, which has substantially improved the clarity and quality of the paper. We hope that the revisions satisfactorily address all comments.

Thank you for your consideration.
